# Supplementary material for: Dietary Patterns and Risk of Chronic Obstructive Pulmonary Disease among Chinese Adults: An 11-Year Prospective Study
Source: Nutrients. 2022 Feb 26;14(5):996. doi: 10.3390/nu14050996 (PMC8912729; doi:10.3390/nu14050996)
Supplement: Supplementary file 1 [file nutrients-14-00996-s001.zip › nutrients-1578067-supplementary.pdf]

Dietary Patterns and Risk of Chronic Obstructive Pulmonary Disease among Chinese Adults: A 11-year Prospective Study

Supplementary Appendix

Table of contents

Members of the China Kadoorie Biobank collaborative group.. 2

Supplemental Table S1. Characteristics of dietary patterns by quintile categories (n = 421,426)... 4

Supplemental Table S2. Comparing dietary patterns constructed from food consumption frequency and estimated amount (n = 421,426). 5

Supplemental Table S3. HRs (95% CIs) for the sensitivity analyses of the association between dietary patterns and risk of COPD. 6

Members of the China Kadoorie Biobank collaborative group:

International Steering Committee: Junshi Chen, Zhengming Chen (PI), Robert Clarke, Rory Collins, Yu Guo, Liming Li (PI), Jun Lv, Richard Peto, Robin Walters. **International Co-ordinating Centre, Oxford:** Daniel Avery, Ruth Boxall, Derrick Bennett, Yumei Chang, Yiping Chen, Zhengming Chen, Robert Clarke, Huaidong Du, Simon Gilbert, Alex Hacker, Mike Hill, Michael Holmes, Andri Iona, Christiana Kartsonaki, Rene Kerosi, Ling Kong, Om Kurmi, Garry Lancaster, Sarah Lewington, Kuang Lin, John McDonnell, Iona Millwood, Qunhua Nie, Jayakrishnan Radhakrishnan, Paul Ryder, Sam Sansome, Dan Schmidt, Paul Sherliker, Rajani Sohoni, Becky Stevens, Iain Turnbull, Robin Walters, Jenny Wang, Lin Wang, Neil Wright, Ling Yang, Xiaoming Yang. **National Co-ordinating Centre, Beijing:** Yu Guo, Xiao Han, Can Hou, Jun Lv, Pei Pei, Chao Liu, Canqing Yu. **10 Regional Co-ordinating Centres: Qingdao CDC:** Zengchang Pang, Ruqin Gao, Shanpeng Li, Shaojie Wang, Yongmei Liu, Ranran Du, Yajing Zang, Liang Cheng, Xiaocao Tian, Hua Zhang, Yaoming Zhai, Feng Ning, Xiaohui Sun, Feifei Li. **Licang CDC:** Silu Lv, Junzheng Wang, Wei Hou. **Heilongjiang Provincial CDC:** Mingyuan Zeng, Ge Jiang, Xue Zhou. **Nangang CDC:** Liqiu Yang, Hui He, Bo Yu, Yanjie Li, Qinai Xu,Quan Kang, Ziyan Guo. **Hainan Provincial CDC:** Dan Wang, Ximin Hu, Jinyan Chen, Yan Fu, Zhenwang Fu, Xiaohuan Wang. **Meilan CDC:** Min Weng, Zhendong Guo, Shukuan Wu,Yilei Li, Huimei Li, Zhifang Fu. **Jiangsu Provincial CDC:** Ming Wu, Yonglin Zhou, Jinyi Zhou, Ran Tao, Jie Yang, Jian Su. **Suzhou CDC:** Fang liu, Jun Zhang, Yihe Hu, Yan Lu, , Liangcai Ma, Aiyu Tang, Shuo Zhang, Jianrong Jin, Jingchao Liu. **Guangxi Provincial CDC:** Zhenzhu Tang, Naying Chen, Ying Huang. **Liuzhou CDC:** Mingqiang Li, Jinhuai Meng, Rong Pan, Qilian Jiang, Jian Lan,Yun Liu, Liuping Wei, Liyuan Zhou, Ningyu Chen Ping Wang, Fanwen Meng, Yulu Qin,, Sisi Wang. **Sichuan Provincial CDC:** Xianping Wu, Ningmei Zhang, Xiaofang Chen,Weiwei Zhou. **Pengzhou CDC:** Guojin Luo, Jianguo Li, Xiaofang Chen, Xunfu Zhong, Jiaqiu Liu, Qiang Sun. **Gansu Provincial CDC:** Pengfei Ge, Xiaolan Ren, Caixia Dong. **Maiji CDC:** Hui Zhang,

Enke Mao, Xiaoping Wang, Tao Wang, Xi zhang. **Henan Provincial CDC:** Ding Zhang, Gang Zhou, Shixian Feng, Liang Chang, Lei Fan. **Huixian CDC:** Yulian Gao, Tianyou He, Huarong Sun, Pan He, Chen Hu, Xukui Zhang, Huifang Wu, Pan He. **Zhejiang Provincial CDC:** Min Yu, Ruying Hu, Hao Wang. Tongxiang CDC: Yijian Qian, Chunmei Wang, Kaixu Xie, Lingli Chen, Yidan Zhang, Dongxia Pan, Qijun Gu. **Hunan Provincial CDC:** Yuelong Huang, Biyun Chen, Li Yin, Huilin Liu, Zhongxi Fu, Qiaohua Xu. **Liuyang CDC:** Xin Xu, Hao Zhang, Huajun Long, Xianzhi Li, Libo Zhang, Zhe Qiu.

Supplemental Table S1. Characteristics of dietary patterns by quintile categories (n = 421,426).

Food or beverage group(g/week)	Traditional northern dietary pattern					Balanced dietary pattern				
	Q1	Q2	Q3	Q4	Q5	Q1	Q2	Q3	Q4	Q5
**Food group, day/week**										
Rice	7.00	6.99	6.93	3.94	1.59	3.72	5.20	5.82	6.08	5.64
Wheat	0.86	1.25	3.06	6.41	6.97	4.24	3.11	3.01	3.44	4.74
Other staple foods	0.26	0.39	0.60	1.06	4.84	2.52	1.59	1.00	0.88	1.16
Meat	4.86	3.76	4.28	3.87	1.84	1.36	2.70	3.86	4.93	5.76
Poultry	1.31	0.82	0.92	0.71	0.20	0.11	0.37	0.73	1.16	1.58
Fish	2.20	1.41	1.61	1.29	0.36	0.20	0.76	1.46	1.93	2.51
Eggs	1.32	1.76	2.92	3.14	3.36	0.96	1.96	2.54	2.83	4.20
Fresh vegetables	6.87	6.90	6.91	6.61	6.87	6.47	6.87	6.91	6.94	6.97
Soybean	1.86	1.67	1.90	1.84	1.21	0.62	1.33	1.71	2.11	2.70
Preserved vegetables	2.68	2.13	2.00	2.57	1.31	1.63	1.95	2.21	2.36	2.55
Fresh fruit	1.91	2.30	3.11	3.51	2.05	0.71	1.29	1.96	3.39	5.53
Dairy products	0.13	0.31	1.13	1.85	1.23	0.04	0.11	0.27	0.79	3.44
**Beverage group, g/week**										
Beer	1.03	1.47	4.13	9.06	5.32	0.08	0.48	1.28	3.82	15.36
Rice wine	14.44	1.18	0.80	0.09	< 0.01	1.71	3.71	4.92	4.17	1.99
Wine	0.06	0.09	0.15	0.16	0.07	< 0.01	< 0.01	0.02	0.07	0.43
Heavy spirit (≥40%)	37.60	24.05	23.79	20.29	11.68	18.98	25.27	26.35	25.17	21.63
Light spirit (<40%)	32.39	4.05	3.04	3.14	6.26	8.57	12.03	12.12	10.40	5.74
Green tea	4.71	5.74	8.22	8.84	4.81	2.18	4.49	6.17	7.72	11.76
Oolong tea	1.04	0.38	0.37	0.09	0.01	0.02	0.11	0.30	0.67	0.78
Black tea	4.44	0.45	0.25	0.04	0.01	1.30	1.45	1.26	0.80	0.38
Other tea	0.02	0.01	0.02	0.01	< 0.01	0.01	0.01	0.01	0.01	0.02

Supplemental Table S2. Comparing dietary patterns constructed from food consumption frequency and estimated amount (n = 421,426).

Traditional northern dietary pattern

		Estimated food consumption amount					Total	kappa	Weighted kappa
		Q5	Q4	Q3	Q2	Q1			
Food consumption frequency	Q1	61,709	21,010	1563	3	0	84,285	0.61	0.79
	Q2	17,497	42,589	24,075	124	0	84,285		
	Q3	5008	20,008	50,193	9077	0	84,286		
	Q4	66	683	8451	63,429	11,656	84,285		
	Q5	0	0	4	11,652	72,629	84,285		
	Total	84,280	84,290	84,286	84,285	84,285	421,426		

Balanced dietary pattern

		Estimated food consumption amount					Total	kappa	Weighted kappa
		Q1	Q2	Q3	Q4	Q5			
Food consumption frequency	Q1	62,171	20,882	1212	0	0	84,265	0.54	0.76
	Q2	20,815	39,719	22,981	790	0	84,305		
	Q3	1280	22,346	41,142	19,396	122	84,286		
	Q4	19	1336	18,697	51,723	12,510	84,285		
	Q5	0	2	254	12,376	71,653	84,285		
	Total	84,285	84,285	84,286	84,285	84,285	421,426		

Note: The results were presented as frequency. The quintile of traditional northern pattern derived from estimated food consumption amount were reversed to match the same direction of food groups.

Supplemental Table S3. HRs (95% CIs) for the sensitivity analyses of the association between dietary patterns and risk of COPD.

	n	Quintile of dietary patterns					*p* for trend
		Q1	Q2	Q3	Q4	Q5	
**Traditional northern dietary pattern**							
**Total**							
Dietary patterns constructed from estimated food amount	421,426	1.00	1.05 (0.99, 1.12)	1.05 (0.97, 1.12)	0.98 (0.87, 1.10)	0.88 (0.75, 1.04)	0.338
Excluding incident cases in first two years	417,647	1.00	1.07 (1.00, 1.13)	1.06 (0.99, 1.13)	1.00 (0.91, 1.11)	0.91 (0.79, 1.06)	0.441
LLN definition for airflow obstruction[a]	414,619	1.00	1.04 (0.98, 1.10)	1.03 (0.97, 1.10)	0.99 (0.90, 1.09)	0.88 (0.76, 1.01)	0.260
Further adjusting for waist circumference[b]	421,426	1.00	1.06 (1.00, 1.12)	1.06 (0.99, 1.12)	1.00 (0.91, 1.10)	0.90 (0.78, 1.03)	0.345
Using propensity scores to adjust for confounders[c]	421,426	1.00	1.10 (1.04, 1.17)	1.08 (1.02, 1.15)	1.01 (0.92, 1.12)	0.90 (0.78, 1.04)	0.404
**Men**							
Dietary patterns constructed from estimated food amount	169,188	1.00	0.99 (0.91, 1.08)	0.94 (0.85, 1.04)	0.96 (0.81, 1.14)	0.99 (0.77, 1.27)	0.554
Excluding incident cases in first two years	167,027	1.00	1.05 (0.96, 1.14)	1.00 (0.92, 1.10)	0.90 (0.78, 1.04)	0.85 (0.69, 1.04)	0.064
LLN definition for airflow obstruction[a]	167,732	1.00	1.02 (0.94, 1.10)	0.98 (0.90, 1.06)	0.88 (0.77, 1.01)	0.82 (0.67, 1.00)	0.030
Further adjusting for waist circumference[b]	169,188	1.00	1.05 (0.97, 1.14)	1.00 (0.92, 1.09)	0.90 (0.79, 1.04)	0.84 (0.69, 1.03)	0.061
Using propensity scores to adjust for confounders[c]	169,188	1.00	1.09 (1.00, 1.18)	1.01 (0.92, 1.10)	0.86 (0.75, 0.99)	0.77 (0.63, 0.94)	0.005
**Women**							
Dietary patterns constructed from estimated food amount	252,238	1.00	1.12 (1.01, 1.25)	1.13 (1.00, 1.26)	1.05 (0.88, 1.24)	0.85 (0.67, 1.08)	0.451
Excluding incident cases in first two years	250,620	1.00	1.08 (0.99, 1.17)	1.13 (1.03, 1.24)	1.17 (1.01, 1.35)	1.05 (0.85, 1.30)	0.148
LLN definition for airflow obstruction[a]	246,887	1.00	1.05 (0.97, 1.14)	1.11 (1.02, 1.22)	1.18 (1.02, 1.36)	1.02 (0.83, 1.25)	0.175
Further adjusting for waist circumference[b]	252,238	1.00	1.07 (0.98, 1.15)	1.13 (1.03, 1.23)	1.19 (1.03, 1.36)	1.04 (0.85, 1.27)	0.112
Using propensity scores to adjust for confounders[c]	252,238	1.00	1.09 (1.01, 1.18)	1.13 (1.04, 1.24)	1.17 (1.01, 1.35)	1.03 (0.83, 1.26)	0.201
Further adjusting for reproductive history[d]	252,196	1.00	1.07 (0.99, 1.16)	1.13 (1.04, 1.24)	1.20 (1.04, 1.37)	1.05 (0.85, 1.29)	0.090
**Balanced dietary pattern**							
**Total**							

	n	Quintile of dietary patterns					*p* for trend
		Q1	Q2	Q3	Q4	Q5	
Dietary patterns constructed from estimated food amount	421,426	1.00	0.91 (0.86, 0.96)	0.88 (0.82, 0.94)	0.83 (0.76, 0.91)	0.76 (0.67, 0.86)	< 0.001
Excluding incident cases in first two years	417,647	1.00	0.89 (0.84, 0.95)	0.88 (0.82, 0.94)	0.86 (0.79, 0.94)	0.78 (0.69, 0.88)	< 0.001
LLN definition for airflow obstruction[a]	414,619	1.00	0.89 (0.84, 0.94)	0.86 (0.81, 0.92)	0.82 (0.76, 0.90)	0.73 (0.65, 0.82)	< 0.001
Further adjusting for waist circumference[b]	421,426	1.00	0.89 (0.84, 0.94)	0.87 (0.82, 0.93)	0.84 (0.77, 0.91)	0.75 (0.67, 0.84)	< 0.001
Using propensity scores to adjust for confounders[c]	421,426	1.00	0.86 (0.81, 0.92)	0.80 (0.74, 0.87)	0.75 (0.68, 0.82)	0.69 (0.61, 0.77)	< 0.001
**Men**							
Dietary patterns constructed from estimated food amount	169,188	1.00	0.95 (0.87, 1.03)	0.93 (0.85, 1.03)	0.90 (0.80, 1.02)	0.84 (0.71, 0.99)	0.043
Excluding incident cases in first two years	167,027	1.00	0.95 (0.87, 1.04)	0.93 (0.84, 1.03)	0.87 (0.77, 0.98)	0.84 (0.71, 0.99)	0.027
LLN definition for airflow obstruction[a]	167,732	1.00	0.94 (0.86, 1.02)	0.89 (0.81, 0.98)	0.83 (0.74, 0.94)	0.79 (0.68, 0.93)	0.002
Further adjusting for waist circumference[b]	169,188	1.00	0.95 (0.87, 1.03)	0.92 (0.83, 1.01)	0.85 (0.75, 0.95)	0.81 (0.69, 0.95)	0.007
Using propensity scores to adjust for confounders[c]	169,188	1.00	0.91 (0.82, 1.00)	0.82 (0.73, 0.92)	0.74 (0.65, 0.84)	0.74 (0.63, 0.87)	< 0.001
**Women**							
Dietary patterns constructed from estimated food amount	252,238	1.00	0.93 (0.86, 1.00)	0.91 (0.83, 1.01)	0.88 (0.77, 1.00)	0.78 (0.64, 0.95)	0.017
Excluding incident cases in first two years	250,620	1.00	0.91 (0.84, 0.99)	0.91 (0.83, 1.01)	0.96 (0.85, 1.09)	0.83 (0.69, 1.00)	0.145
LLN definition for airflow obstruction[a]	246,887	1.00	0.91 (0.84, 0.99)	0.91 (0.82, 1.00)	0.91 (0.80, 1.03)	0.77 (0.64, 0.92)	0.014
Further adjusting for waist circumference[b]	252,238	1.00	0.90 (0.84, 0.98)	0.90 (0.82, 0.99)	0.92 (0.82, 1.04)	0.79 (0.66, 0.94)	0.033
Using propensity scores to adjust for confounders[c]	252,238	1.00	0.87 (0.80, 0.95)	0.87 (0.77, 0.97)	0.90 (0.78, 1.03)	0.78 (0.65, 0.94)	0.024
Further adjusting for reproductive history[d]	252,196	1.00	0.90 (0.83, 0.98)	0.90 (0.82, 0.99)	0.92 (0.82, 1.04)	0.79 (0.66, 0.94)	0.033

Note: Results were based on model 3, adjusting sex (male or female, only in total population), education level (no formal school, primary school, middle school, high school, or college/university), marital status (married or other), household income (< 10,000, 10,000-19,999, or ≥ 20,000 Chinese yuan/year), tobacco smoking (never/occasional, former and having quit ≥ 5 years or < 5 years, current and 1-14 cigarettes/day, 15-24 cigarettes/day, or ≥ 25 cigarettes/day), alcohol consumption (not weekly drinking, ex-regular drinkers, not daily, daily and < 15 g/day, 15-29 g/day, 30-59 g/day, or ≥ 60 g/day), BMI (continuous, kg/m^2), physical activity (continuous, MET-h), nutritional supplement (yes or no), daily energy intake (continuous in log-transformed formal, kJ/day), passive smoking (never lived with a smoker, lived with a smoker for < 20 years, lived with a smoker for ≥ 20

	n	Quintile of dietary patterns					p for trend
		Q1	Q2	Q3	Q4	Q5	

years and exposure < 20 h/week, or lived with a smoker for ≥ 20 years and exposure ≥ 20 h/week), cook fuel pollution (never or occasionally cook, daily cook with clean fuel, daily cook with solid fuel, or daily cook with other fuel), and heat fuel pollution (never or occasionally heat, heat with clean fuel, heat with solid fuel, or heat with other fuel).

HR = hazard ratio; CI = confidence interval; LLN = lower limit of normal; BMI = body mass index; MET-h = metabolic equivalent task-hour.

[a] Excluding participants with airflow obstruction at baseline using LLN definition rather than FEV1/FVC <0.7.

[b] Additionally adjusting for waist circumference (male: < 700, 700-849, 850-899, 900-949, or ≥ 950 mm; female: < 650, 650-799, 800-849, 850-899, or ≥ 900 mm).

[c] Propensity scores were estimated by multinomial logistic models.

[d] 44 women were with a missing or abnormal value of reproductive history. Reproductive history included menopause status (premenopausal, perimenopausal or postmenopausal), the use of oral contraceptive pills (never, past use, or current use), the history of pregnancy (the number of live births, still births, spontaneous abortion and induced abortion), and the history of gynecological surgery (hysterectomy, oophorectomy or mastectomy).
